# Phylogenetic and evolutionary analysis of foot-and-mouth disease virus A/ASIA/Sea-97 lineage

**DOI:** 10.1007/s11262-021-01848-7

**Published:** 2021-07-14

**Authors:** Soyeon Bae, Vladimir Li, Juyong Hong, Jin Nam Kim, Heebal Kim

**Affiliations:** 1 Department of Agricultural Biotechnology and Research Institute of Agriculture and Life Sciences, Seoul National University, Seoul, 08826 Republic of Korea; 2 Interdisciplinary Program in Bioinformatics, Seoul National University, Seoul, 08826 Republic of Korea; 3 eGnome, Inc, Seoul, Republic of Korea

**Keywords:** Foot-and-mouth disease virus, Phylogenetic analysis, Bayesian analysis, Virus evolution

## Abstract

Foot-and-mouth disease virus (FMDV) A/ASIA/Sea-97 is a predominant lineage in Southeast Asia and East Asia. However, Sea-97 lineage has not been well studied since its first outbreak in Thailand in 1997. Thus, we conducted phylogenetic and evolutionary analysis of Sea-97 using 224 VP1 sequences of FMDV A/ASIA during 1960 and 2018. Phylogenetic analysis revealed that Sea-97 lineage can be classified into five groups (G1–G5). After the emergence of G2 from G1, the genetic diversity of Sea-97 increased sharply, causing divergence into G3, G4 and G5. During this evolutionary process, Sea-97 lineage, which was initially found only in some countries in Southeast Asia, gradually spread to East Asia. The evolution rate of this lineage was estimated to be 1.2 × 10^–2^ substitutions/site/year and there were many differences in amino acid residues compared to vaccine strain. Substitutions at antigenically important sites may affect the efficacy of the vaccine, suggesting the need for appropriate vaccine strains. Our results could provide meaningful information to understand comprehensive characteristic of Sea-97 lineage.

**Supplementary Information:**

The online version contains supplementary material available at 10.1007/s11262-021-01848-7.

## Introduction

Foot-and-mouth disease (FMD), a contagious disease that affects cloven-hoofed animals, is caused by FMD virus (FMDV), a member of the *Aphthovirus* genus within the *Picornaviridae* family [1]. There are seven distinct types of FMDV serotypes: O, A, C, Asia 1, SAT 1, SAT 2, and SAT 3. Among them, serotype A is widely distributed and considered to be highly genetically and antigenically diverse, making vaccination control difficult [2, 3].

The nucleotide sequence encoding VP1, one of the structural proteins constituting the capsid, is used for characterization and phylogenetic analysis of FMDV [2, 4]. Based on the analysis of VP1 sequences, serotype A was classified into 26 genotypes [5]. 11 out of the 26 genotypes belonged to the ASIA topotype, and three of them seem to be the major lineages until recently. Several subtypes of A/ASIA/Iran-05 and G-VII were identified, and many studies were conducted on them [3, 6–13]. However, although A/ASIA/Sea-97 was reported in various countries in East Asia and Southeast Asia [14], only a few studies were conducted using a small number of sequence data [15, 16].

In this study, we investigated the phylogeny and evolution of Sea-97 lineage. All publicly available FMDV A/ASIA VP1 sequences were collected and used to reconstruct the phylogenetic tree. We subdivided Sea-97 into five groups based on the phylogeny and analyzed their phylodynamics.

## Materials and methods

We collected VP1 coding region, polyprotein, and full genome sequences of FMDV A/ASIA from GenBank (www.ncbi.nlm.nih.gov). All nucleotide and protein sequences were aligned using MAFFT v7.419 and trimmed manually using MEGA X [17, 18]. Based on aligned protein sequences, multiple codon alignment was performed using PAL2NAL web server [19]. The final dataset contained 224 VP1 sequences of 633 bp isolated from eighteen countries between 1960 and 2018. The GenBank accession numbers are provided in Table S1.

Pairwise distances between 224 VP1 sequences were calculated and an unweighted pair group mean average (UPGMA) tree was constructed using DNADIST and NEIGHBOR in PHYLIP package v3.698 [20]. The F84 model was used to compute the distance matrix, which assumed unequal base frequencies and different transition and transversion rates. The resulting tree was visualized in FigTree v1.4.4 [21]. Then, we distinguished nine lineages based on the prototype strains specified in FAO World Reference Laboratory for FMD (WRLFMD) [22].

Bayesian evolutionary analysis was performed using BEAST v2.6.3 [23]. The best-fit nucleotide substitution model was determined using ModelTest-NG [24]. We used relaxed clock log normal and coalescent Bayesian skyline model as a prior. Four independent Markov chain Monte Carlo (MCMC) chains were run for 50 million steps, sampled every 5,000 steps, and then combined using LogCombiner [23]. The first 10% of chain in each run were discarded as burn-in. We analyzed the MCMC output log file using Tracer v1.7.1 [25]. We construct the maximum clade credibility (MCC) tree using TreeAnnotator [23].

Then, we extracted VP1 sequences of Sea-97 and conducted Bayesian analysis (two chains for 50 million MCMC iterations, sampled every 5000 steps). We inferred phylogeographical history of Sea-97 and constructed the corresponding Bayesian tree. The MCC tree with location traits was visualized using SpreadD3 v0.9.6 [26]. Bayesian skyline plot (BSP) was reconstructed in Tracer v1.7.1 [25].

We investigated selection pressures on VP1 gene of Sea-97 using Datamonkey 2.0 webserver [27]. Fixed Effects Likelihood (FEL), Fast, Unconstrained Bayesian AppRoximation (FUBAR), Single-Likelihood Ancestor Counting (SLAC) methods, and Mixed Effects Model of Evolution (MEME) were used to detect sites under pervasive and episodic positive selections [28–30].

## Results and discussion

**Fig. 1:**
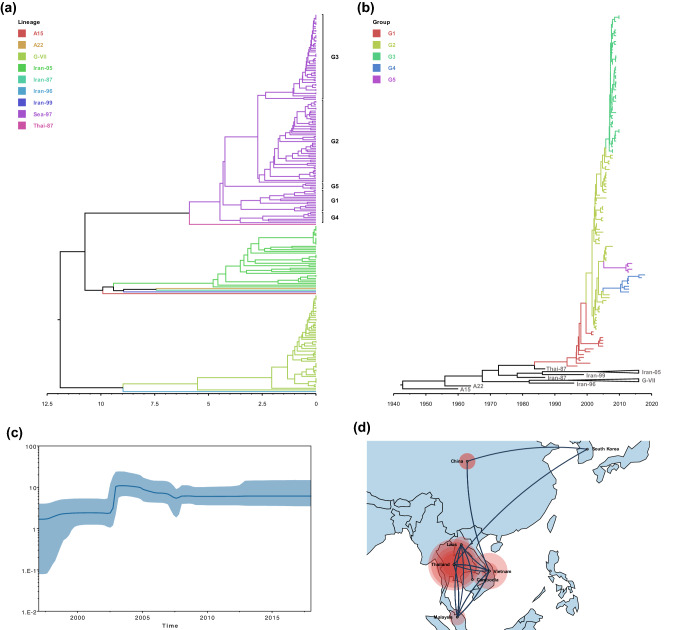
**a** UPGMA tree derived from 224 VP1 nucleotide sequences of FMDV A/ASIA. The *x*-axis represents the %ND. Branches are colored according to the lineage. **b** MCC tree derived from 224 VP1 nucleotide sequences of FMDV A/ASIA. The *x*-axis represents the year. Branches are colored according to the lineage and branches of G-VII and Iran-05 are collapsed. **c** BSP of A/ASIA/Sea-97. The *x*-axis and *y*-axis represent the year and the effective population size, respectively. The thick blue line indicates median effective population size, and the light blue region means their 95% HPD interval. **d** Spatial distribution of A/ASIA/Sea-97. The lines show the transmission between locations. The size of the red circles is proportional to the intensity of the virus presence in that region

In general, when classifying the subtype of FMDV, the UPGMA tree is constructed using the VP1 sequences and the percent nucleotide divergence (ND) is measured [5, 31–33]. The threshold for classifying the sub-lineage is not clearly established, but it seems that lineages can be divided if they show at least 2.7–3.5% ND [34]. Figure 1a shows the UPGMA tree for 224 VP1 sequences of ASIA topotype. A15, Thai-87, and Sea-97 were isolated in East Asia and Southeast Asia countries, and only Sea-97 appears to be circulating at present time. As shown in the UPGMA tree, Sea-97 was clustered into five major groups denoted G1 to G5 (Table 1). G4 was distinguished from the other groups of Sea-97 at 4.5% ND. G1 and G5 were separated at the ND of 4.3% and 4.2%, respectively. The clades of G2 and G3 were divided at 2.7% ND. WRLFMD prototype strains of Sea-97 (A/TAI/2/97 and A/TAI/7/2003) belonged to G1 group. G1, G2 and G5 only included isolates from Southeast Asia, but G3 and G4 expanded to East Asia.

**Table 1:** The summary of groups of FMDV A/ASIA/Sea-97 classified based on UPGMA tree

Group	Isolates	Year	Country
G1	13	1997–2005	Thailand, Malaysia
G2	49	2003–2008	Cambodia, Laos, Malaysia, Thailand, Vietnam
G3	51	2008–2010	China, Laos, Malaysia, South Korea, Thailand, Vietnam
G4	7	2013–2018	China, Malaysia, South Korea, Vietnam
G5	4	2013–2014	Laos, Malaysia, Thailand, Vietnam

The MCC tree for VP1 sequences of the ASIA topotype is shown in Fig. 1b. The most recent common ancestor (MRCA) of Sea-97 was dated to be around 1993 [95% HPD = 1990.3525–1996.5827]. Then, G2 emerged from G1 in 2001.5275 [95% HPD = 2000.5886–2002.3205], and G3, G4, and G5 emerged from G2 in 2005.7843 [95% HPD = 2004.9717–2006.5114], 2010.395 [95% HPD = 2008.8512–2011.5133], and 2012.0403 [95% HPD = 2011.2336–2012.7064], respectively.

A set of 124 VP1 sequences of Sea-97 was further analyzed to infer evolutionary history. The mean evolutionary rate of Sea-97 ($1.2\times {10}^{-2}$ s/s/y [95% HPD = $9.29\times {10}^{-3}-1.51\times {10}^{-2}$]) was estimated to be much greater than that of global FMDV serotype A samples ($4.26\times {10}^{-3}$ [35] and $5.77\times {10}^{-3}$ [36] s/s/y), and it was similar to the rates of other lineages of topotype ASIA ($1.25\times {10}^{-2}$ for Iran-05 [7] and $1.1\times {10}^{-2}$ for G-VII clade C [13] s/s/y). The BSP showed that the genetic diversity of Sea-97 isolates was constant until 2001, then increased sharply around 2002–2003, and then remained constant again (Fig. 1c). This pattern of population size is probably due to the emergence of G2 around 2001 and subsequent emergences of G3, G4, and G5. The reconstructed spatial diffusion of Sea-97 showed that this lineage first occurred in Thailand and spread to Malaysia and neighboring countries, and then from Vietnam to China and later to South Korea (Fig. 1d). It seems that this lineage has been actively circulating within Southeast Asian countries.

Amino acid sequences of each group of Sea-97 were compared with the sequence of A22 (A22/IRQ/24/64), one of the widely used vaccine strains [37]. The sequence logo of each group obtained using Jalview v2.11.1.3 [38] was used for comparison (Fig. S1). The length of VP1 sequence of A22 was 211 amino acids and there was a gap at position 140 in Sea-97. Positions 43–45, 83, 96, 141–160 (G-H loop), 169–173 and 200–211 (C-terminus) of VP1 were previously suggested as candidate regions that may affect antigenic properties of FMDV serotype A [9, 39–44]. Compared to A22, Sea-97 had many substitutions at these residues (Q43K, N44P, L45V, D83T/A, T141E/G/V/A/Q, G142T/N/P/S, P149S, V154I/L, A160T, T171E, H173Q/R, H201Y and K204R). Most of sites under positive selection also belonged to these candidate regions (Table S2). In particular, Q43K, N44P, D81T/A, T141E/G/V/A, G142T/N/S, P149S, A160T, T171E, H173Q and H201Y have been substituted with amino acids with different biochemical properties. Therefore, it has the potential to have a major impact on antigenic properties. There is already a study suggesting that P149 rather than S149 matches well with the A22 vaccine [43]. It seems necessary to test whether these substitutions actually affect the efficacy of the currently used vaccine. If the existing vaccine does not match Sea-97 well, an appropriate vaccine for this lineage needs to be developed.

This study provides information to understand comprehensive characteristic of the Sea-97 lineage. However, the number of sequences available in the current public database is insufficient. Although the number of recent sequence data are small, our findings could provide meaningful basic information on strategies to control FMDV in Asia. Based on our results, it seems necessary to collect more data and perform an extended analysis in future studies.

## Supplementary Information

Below is the link to the electronic supplementary material.

Supplementary file 1 (PDF 455 kb)

Supplementary file 2 (DOCX 44 kb)

Supplementary file 3 (DOCX 17 kb)

## Data Availability

Not applicable (No datasets were generated during the current study. All data analysed during this study were derived from GenBank database. Accession numbers are included in supplementary information files.).

## References

[1] Grubman MJ, Baxt B (2004). Foot-and-Mouth Disease. Clin Microbiol Rev.

[2] Knowles N, Samuel A (2003). Molecular epidemiology of foot-and-mouth disease virus. Virus Res.

[3] Jangra RK, Tosh C, Sanyal A, Hemadri D, Bandyopadhyay SK (2005). Antigenic and genetic analyses of foot-and-mouth disease virus type A isolates for selection of candidate vaccine strain reveals emergence of a variant virus that is responsible for most recent outbreaks in India. Virus Res.

[4] Carrillo C, Tulman E, Delhon G, Lu Z, Carreno A, Vagnozzi A, Kutish G, Rock D (2005). Comparative genomics of foot-and-mouth disease virus. J Virol.

[5] Mohapatra JK, Subramaniam S, Pandey LK, Pawar SS, De A, Das B, Sanyal A, Pattnaik B (2011). Phylogenetic structure of serotype A foot-and-mouth disease virus: global diversity and the Indian perspective. J Gen Virol.

[6] Knowles N, Nazem Shirazi M, Wadsworth J, Swabey K, Stirling J, Statham R, Li Y, Hutchings G, Ferris N, Parlak Ü (2009). Recent spread of a new strain (A-Iran-05) of foot-and-mouth disease virus type A in the Middle East. Transbound Emerg Dis.

[7] Jamal SM, Ferrari G, Ahmed S, Normann P, Curry S, Belsham GJ (2011). Evolutionary analysis of serotype A foot-and-mouth disease viruses circulating in Pakistan and Afghanistan during 2002–2009. J Gen Virol.

[8] Upadhyaya S, Ayelet G, Paul G, King DP, Paton DJ, Mahapatra M (2014). Genetic basis of antigenic variation in foot-and-mouth disease serotype A viruses from the middle east. Vaccine.

[9] Mahapatra M, Statham B, Li Y, Hammond J, Paton D, Parida S (2016). Emergence of antigenic variants within serotype A FMDV in the middle east with antigenically critical amino acid substitutions. Vaccine.

[10] Ullah A, Jamal S, Romey A, Gorna K, Kakar M, Abbas F, Ahmad J, Zientara S, Bakkali Kassimi L (2017). Genetic characterization of serotypes A and Asia-1 foot-and-mouth disease viruses in Balochistan, Pakistan, in 2011. Transbound Emerg Dis.

[11] Jamal SM, Belsham GJ (2018). Molecular epidemiology, evolution and phylogeny of foot-and-mouth disease virus. Infect Genet Evol.

[12] Das B, Mohapatra JK, Pande V, Subramaniam S, Sanyal A (2016). Evolution of foot-and-mouth disease virus serotype A capsid coding (P1) region on a timescale of three decades in an endemic context. Infect Genet Evol.

[13] Bachanek-Bankowska K, Di Nardo A, Wadsworth J, Henry EK, Parlak Ü, Timina A, Mischenko A, Qasim IA, Abdollahi D, Sultana M (2018). Foot-and-mouth disease in the middle east caused by an A/ASIA/G-VII virus lineage, 2015–2016. Emerg Infect Dis.

[14] Brito B, Rodriguez L, Hammond J, Pinto J, Perez A (2017). Review of the global distribution of foot-and-mouth disease virus from 2007 to 2014. Transbound Emerg Dis.

[15] Nguyen T, Lee K-N, Ko Y-J, Lee H-S, Nguyen VC, Mai TD, Do TH, Kim S-M, Cho I-S, Park J-H (2010). Molecular characterization of serotype A foot-and-mouth disease viruses circulating in Vietnam in 2009. Vet Microbiol.

[16] Vu TTH, Duong H-Q, Song D (2016). Evolutionary phylodynamics of foot-and-mouth disease virus serotypes O and A circulating in Vietnam. BMC Vet Res.

[17] Katoh K, Standley DM (2013). MAFFT multiple sequence alignment software version 7: improvements in performance and usability. Mol Biol Evol.

[18] Kumar S, Stecher G, Li M, Knyaz C, Tamura K (2018). MEGA X: molecular evolutionary genetics analysis across computing platforms. Mol Biol Evol.

[19] Suyama M, Torrents D, Bork P (2006). PAL2NAL: robust conversion of protein sequence alignments into the corresponding codon alignments. Nucleic Acids Res.

[20] Felsenstein J (2005) PHYLIP (Phylogeny Inference Package) version 3.6. http://www.evolution.gs.washington.edu/phylip.html. Accessed 3 December 2020

[21] Rambaut A FigTree v1.4.4. http://tree.bio.ed.ac.uk/software/figtree/. Accessed 3 December 2020

[22] Knowles NJ, Wadsworth J, Bachanek-Bankowska K, King D (2016). VP1 sequencing protocol for foot and mouth disease virus molecular epidemiology. Rev Sci Tech.

[23] Bouckaert R, Vaughan TG, Barido-Sottani J, Duchêne S, Fourment M, Gavryushkina A, Heled J, Jones G, Kühnert D, De Maio N (2019). BEAST 2.5: An advanced software platform for Bayesian evolutionary analysis. PLoS Comput Biol.

[24] Darriba D, Posada D, Kozlov AM, Stamatakis A, Morel B, Flouri T (2020). ModelTest-NG: a new and scalable tool for the selection of DNA and protein evolutionary models. Mol Biol Evol.

[25] Rambaut A, Drummond AJ, Xie D, Baele G, Suchard MA (2018). Posterior summarization in Bayesian phylogenetics using Tracer 1.7. Syst Biol.

[26] Bielejec F, Baele G, Vrancken B, Suchard MA, Rambaut A, Lemey P (2016). SpreaD3: interactive visualization of spatiotemporal history and trait evolutionary processes. Mol Biol Evol.

[27] Weaver S, Shank SD, Spielman SJ, Li M, Muse SV, Kosakovsky Pond SL (2018). Datamonkey 2.0: a modern web application for characterizing selective and other evolutionary processes. Mol Biol Evol.

[28] Kosakovsky Pond SL, Frost SD (2005). Not so different after all: a comparison of methods for detecting amino acid sites under selection. Mol Biol Evol.

[29] Murrell B, Moola S, Mabona A, Weighill T, Sheward D, Kosakovsky Pond SL, Scheffler K (2013). FUBAR: a fast, unconstrained bayesian approximation for inferring selection. Mol Biol Evol.

[30] Murrell B, Wertheim JO, Moola S, Weighill T, Scheffler K, Pond SLK (2012). Detecting individual sites subject to episodic diversifying selection. PLoS Genet.

[31] Samuel A, Knowles N (2001). Foot-and-mouth disease type O viruses exhibit genetically and geographically distinct evolutionary lineages (topotypes). J Gen Virol.

[32] Abeyratne S, Amarasekera S, Ranaweera L, Salpadoru T, Thilakarathne S, Knowles N, Wadsworth J, Puvanendiran S, Kothalawala H, Jayathilake B (2018). The phylogenetic analysis of VP1 genomic region in foot-and-mouth disease virus serotype O isolates in Sri Lanka reveals the existence of'Srl-97', a newly named endemic lineage. PLoS ONE.

[33] Ranaweera LT, Wijesundara UK, Jayarathne HS-M, Knowles N, Wadsworth J, Mioulet V, Adikari J, Weebadde C, Sooriyapathirana SS (2019). Characterization of the FMDV-serotype-O isolates collected during 1962 and 1997 discloses new topotypes, CEY-1 and WCSA-1, and six new lineages. Sci Rep.

[34] Mishu ID, Akter S, Alam A, Hossain MA, Sultana M (2020). In silico evolutionary divergence analysis suggests the potentiality of capsid protein VP2 in serotype-independent foot-and-mouth disease virus detection. Front Vet Sci.

[35] Tully DC, Fares MA (2008). The tale of a modern animal plague: tracing the evolutionary history and determining the time-scale for foot and mouth disease virus. Virology.

[36] Yoon SH, Lee K-N, Park J-H, Kim H (2011). Molecular epidemiology of foot-and-mouth disease virus serotypes A and O with emphasis on Korean isolates: temporal and spatial dynamics. Arch Virol.

[37] Mahapatra M, Parida S (2018). Foot and mouth disease vaccine strain selection: current approaches and future perspectives. Expert Rev Vaccines.

[38] Waterhouse AM, Procter JB, Martin DM, Clamp M, Barton GJ (2009). Jalview Version 2—a multiple sequence alignment editor and analysis workbench. Bioinformatics.

[39] Thomas A, Woortmeijer R, Puijk W, Barteling S (1988). Antigenic sites on foot-and-mouth disease virus type A10. J Virol.

[40] Baxt B, Vakharia V, Moore D, Franke A, Morgan D (1989). Analysis of neutralizing antigenic sites on the surface of type A12 foot-and-mouth disease virus. J Virol.

[41] Bolwell C, Clarke B, Parry N, Ouldridge E, Brown F, Rowlands D (1989). Epitope mapping of foot-and-mouth disease virus with neutralizing monoclonal antibodies. J Gen Virol.

[42] Mahapatra M, Seki C, Upadhyaya S, Barnett PV, La Torre J, Paton D (2011). Characterisation and epitope mapping of neutralising monoclonal antibodies to A24 Cruzeiro strain of FMDV. Vet Microbiol.

[43] Ludi AB, Horton D, Li Y, Mahapatra M, King D, Knowles N, Russell C, Paton D, Wood J, Smith DJ (2014). Antigenic variation of foot-and-mouth disease virus serotype A. J Gen Virol.

[44] Bari FD, Parida S, Asfor AS, Haydon DT, Reeve R, Paton DJ, Mahapatra M (2015). Prediction and characterization of novel epitopes of serotype A foot-and-mouth disease viruses circulating in East Africa using site-directed mutagenesis. J Gen Virol.

