# Supplementary material for: Phylogenetic and evolutionary analysis of foot-and-mouth disease virus A/ASIA/Sea-97 lineage
Source: Virus Genes. 2021 Jul 14;57(5):443–7. doi: 10.1007/s11262-021-01848-7 (PMC8445868; doi:10.1007/s11262-021-01848-7)
Supplement: Supplementary file 1 — (PDF 455 kb) [file 11262_2021_1848_MOESM1_ESM.pdf]

Phylogenetic and Evolutionary Analysis of Foot-and-Mouth Disease Virus A/ASIA/Sea-97 Lineage

Soyeon Bae[1], Vladimir Li[2], Juyong Hong[1], Jin Nam Kim[3] and Heebal Kim[1,2,3,*]

[1]Department of Agricultural Biotechnology and Research Institute of Agriculture and Life Sciences, Seoul National University, Seoul 08826, Republic of Korea.

[2]Interdisciplinary Program in Bioinformatics, Seoul National University, Seoul 08826, Republic of Korea.

[3]eGnome, Inc, Seoul, Republic of Korea.

[*]Corresponding Author: Heebal Kim heebal@snu.ac.kr

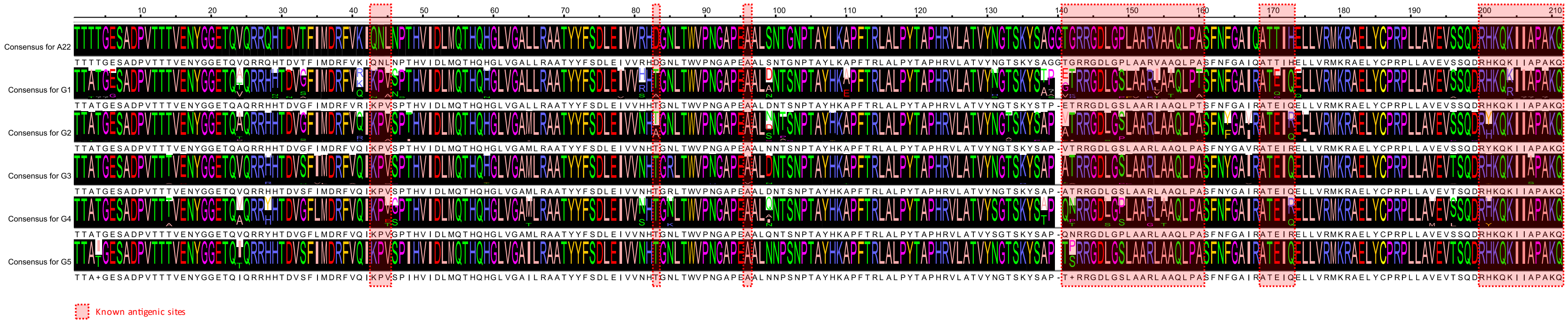

Fig. S1 Normalized sequence logo and consensus sequence of each group of Sea-97
