# Supplementary material for: Phylogenetic and evolutionary analysis of foot-and-mouth disease virus A/ASIA/Sea-97 lineage
Source: Virus Genes. 2021 Jul 14;57(5):443–7. doi: 10.1007/s11262-021-01848-7 (PMC8445868; doi:10.1007/s11262-021-01848-7)
Supplement: Supplementary file 2 — (DOCX 44 kb) [file 11262_2021_1848_MOESM2_ESM.docx]

**Phylogenetic and Evolutionary Analysis of Foot-and-Mouth Disease Virus A/ASIA/Sea-97 Lineage**

Soyeon Bae^1^, Vladimir Li^2^, Juyong Hong^1^, Jin Nam Kim^3^ and Heebal Kim^1,2,3,*^

^1^Department of Agricultural Biotechnology and Research Institute of Agriculture and Life Sciences, Seoul National University, Seoul 08826, Republic of Korea.

^2^Interdisciplinary Program in Bioinformatics, Seoul National University, Seoul 08826, Republic of Korea.

^3^eGnome, Inc, Seoul, Republic of Korea.

^*^Corresponding Author: Heebal Kim [heebal@snu.ac.kr](mailto:heebal@snu.ac.kr)

**Table S1** FMDV/A sequences used in this study

| **GenBank accession** | **Isolate** | **Lineage** | **Year** | **Country** | **Host** |
| --- | --- | --- | --- | --- | --- |
| AF390646 | A/IND/40/2000 | G-VII | 1999 | India | cattle |
| AY593755 | A15/Bangkok/TAI/60 | A15 | 1960 | Thailand | cattle |
| AY593763 | A22/IRQ/24/64 | A22 | 1964 | Iraq | cattle |
| EF208769 | A/IRN/1/2005 | Iran-05 | 2005 | Iran | cattle |
| EF208770 | A/IRN/2/87 | Iran-87 | 1987 | Iran | cattle |
| EF208771 | A/IRN/1/96 | Iran-96 | 1996 | Iran | cattle |
| EF208772 | A/IRN/22/99 | Iran-99 | 1999 | Azerbaijan | cattle |
| EF208777 | A/TAI/118/87 | Thai-87 | 1987 | Thailand | - |
| EF208778 | A/TAI/2/97 | Sea-97 (G1) | 1997 | Thailand | - |
| EU414533 | A/MAY/2/2002 | Sea-97 (G1) | 2002 | Malaysia | cattle |
| EU667455 | A/LAO/36/2003 | Sea-97 (G2) | 2003 | Laos | cattle |
| EU667456 | A/LAO/1/2006 | Sea-97 (G2) | 2006 | Laos | cattle |
| EU667457 | A/LAO/6/2006 | Sea-97 (G2) | 2006 | Laos | cattle |
| EU667458 | A/LAO/7/2006 | Sea-97 (G2) | 2006 | Laos | cattle |
| EU667459 | A/LAO/8/2006 | Sea-97 (G2) | 2006 | Laos | cattle |
| FJ755007 | A/AFG/6/2007 | Iran-05 | 2007 | Afghanistan | cattle |
| FJ755010 | A/BAR/6/2008 | Iran-05 | 2008 | Bahrain | cattle |
| FJ755133 | A/TUR/1/2008 | Iran-05 | 2008 | Turkey | cattle |
| FJ755155 | A/TUR/33/2008 | Iran-05 | 2008 | Turkey | cattle |
| GQ406247 | A/VN/09/2009 | Sea-97 (G3) | 2009 | Vietnam | cattle |
| GQ406248 | A/VN/02/2009 | Sea-97 (G3) | 2009 | Vietnam | cattle |
| GQ406249 | A/VN/03/2009 | Sea-97 (G3) | 2009 | Vietnam | cattle |
| GQ406250 | A/VN/11/2009 | Sea-97 (G3) | 2009 | Vietnam | cattle |
| GQ406251 | A/VN/16/2009 | Sea-97 (G3) | 2009 | Vietnam | pig |
| GQ406252 | A/VN/20/2009 | Sea-97 (G3) | 2009 | Vietnam | buffalo |
| GU441855 | A/Pocheon/KOR/2010 | Sea-97 (G3) | 2010 | South Korea | cattle |
| GU582091 | A/VN/14/2009 | Sea-97 (G3) | 2009 | Vietnam | buffalo |
| HQ116292 | A/CAM/1/2006 | Sea-97 (G2) | 2006 | Cambodia | cattle |
| HQ116293 | A/CAM/2/2006 | Sea-97 (G2) | 2006 | Cambodia | cattle |
| HQ116294 | A/CAM/2/2008 | Sea-97 (G2) | 2008 | Cambodia | cattle |
| HQ116295 | A/LAO/4/2008 | Sea-97 (G3) | 2008 | Laos | cattle |
| HQ116296 | A/MAY/1/2003 | Sea-97 (G2) | 2003 | Malaysia | cattle |
| HQ116297 | A/MAY/3/2003 | Sea-97 (G2) | 2003 | Malaysia | cattle |
| HQ116298 | A/MAY/4/2003 | Sea-97 (G2) | 2003 | Malaysia | cattle |
| HQ116299 | A/MAY/5/2003 | Sea-97 (G2) | 2003 | Malaysia | cattle |
| HQ116300 | A/MAY/5/2005 | Sea-97 (G1) | 2005 | Malaysia | cattle |
| HQ116301 | A/MAY/1/2007 | Sea-97 (G2) | 2007 | Malaysia | pig |
| HQ116302 | A/MAY/3/2007 | Sea-97 (G2) | 2007 | Malaysia | cattle |
| HQ116303 | A/MAY/1/2008 | Sea-97 (G3) | 2008 | Malaysia | cattle |
| HQ116304 | A/MAY/3/2008 | Sea-97 (G3) | 2008 | Malaysia | cattle |
| HQ116305 | A/MAY/2/2009 | Sea-97 (G3) | 2009 | Malaysia | cattle |
| HQ116306 | A/MAY/9/2009 | Sea-97 (G3) | 2009 | Malaysia | cattle |
| HQ116307 | A/TAI/1/2001 | Sea-97 (G1) | 2001 | Thailand | cattle |
| HQ116308 | A/TAI/3/2001 | Sea-97 (G1) | 2001 | Thailand | cattle |
| HQ116309 | A/TAI/2/2002 | Sea-97 (G1) | 2002 | Thailand | cattle |
| HQ116310 | A/TAI/7/2002 | Sea-97 (G1) | 2002 | Thailand | pig |
| HQ116311 | A/TAI/4/2003 | Sea-97 (G2) | 2003 | Thailand | cattle |
| HQ116312 | A/TAI/7/2003 | Sea-97 (G2) | 2003 | Thailand | cattle |
| HQ116313 | A/TAI/8/2003 | Sea-97 (G2) | 2003 | Thailand | pig |
| HQ116314 | A/TAI/10/2003 | Sea-97 (G2) | 2003 | Thailand | cattle |
| HQ116315 | A/TAI/11/2003 | Sea-97 (G2) | 2003 | Thailand | cattle |
| HQ116316 | A/TAI/12/2003 | Sea-97 (G2) | 2003 | Thailand | cattle |
| HQ116317 | A/TAI/6/2004 | Sea-97 (G2) | 2004 | Thailand | cattle |
| HQ116318 | A/TAI/9/2004 | Sea-97 (G2) | 2004 | Thailand | cattle |
| HQ116319 | A/TAI/2/2005 | Sea-97 (G1) | 2005 | Thailand | cattle |
| HQ116320 | A/TAI/3/2005 | Sea-97 (G2) | 2005 | Thailand | cattle |
| HQ116321 | A/TAI/4/2005 | Sea-97 (G1) | 2005 | Thailand | cattle |
| HQ116322 | A/TAI/7/2005 | Sea-97 (G1) | 2005 | Thailand | cattle |
| HQ116323 | A/TAI/11/2005 | Sea-97 (G2) | 2005 | Thailand | cattle |
| HQ116324 | A/TAI/1/2006 | Sea-97 (G2) | 2006 | Thailand | cattle |
| HQ116325 | A/TAI/2/2006 | Sea-97 (G2) | 2006 | Thailand | cattle |
| HQ116326 | A/TAI/3/2006 | Sea-97 (G2) | 2006 | Thailand | cattle |
| HQ116327 | A/TAI/4/2006 | Sea-97 (G2) | 2006 | Thailand | cattle |
| HQ116328 | A/TAI/6/2006 | Sea-97 (G2) | 2006 | Thailand | cattle |
| HQ116329 | A/TAI/7/2006 | Sea-97 (G2) | 2006 | Thailand | cattle |
| HQ116330 | A/TAI/8/2006 | Sea-97 (G2) | 2006 | Thailand | cattle |
| HQ116331 | A/TAI/9/2006 | Sea-97 (G2) | 2006 | Thailand | cattle |
| HQ116332 | A/TAI/10/2006 | Sea-97 (G2) | 2006 | Thailand | cattle |
| HQ116333 | A/TAI/11/2006 | Sea-97 (G2) | 2006 | Thailand | cattle |
| HQ116334 | A/TAI/3/2007 | Sea-97 (G2) | 2007 | Thailand | cattle |
| HQ116335 | A/TAI/11/2007 | Sea-97 (G2) | 2007 | Thailand | cattle |
| HQ116336 | A/TAI/4/2008 | Sea-97 (G3) | 2008 | Thailand | cattle |
| HQ116337 | A/TAI/8/2008 | Sea-97 (G3) | 2008 | Thailand | cattle |
| HQ116338 | A/TAI/9/2008 | Sea-97 (G3) | 2008 | Thailand | buffalo |
| HQ116339 | A/TAI/10/2008 | Sea-97 (G3) | 2008 | Thailand | cattle |
| HQ116340 | A/TAI/11/2008 | Sea-97 (G3) | 2008 | Thailand | cattle |
| HQ116341 | A/TAI/13/2008 | Sea-97 (G3) | 2008 | Thailand | cattle |
| HQ116342 | A/TAI/14/2008 | Sea-97 (G3) | 2008 | Thailand | cattle |
| HQ116343 | A/TAI/15/2008 | Sea-97 (G3) | 2008 | Thailand | cattle |
| HQ116344 | A/TAI/16/2008 | Sea-97 (G3) | 2008 | Thailand | cattle |
| HQ116345 | A/TAI/17/2008 | Sea-97 (G3) | 2008 | Thailand | cattle |
| HQ116346 | A/TAI/18/2008 | Sea-97 (G3) | 2008 | Thailand | cattle |
| HQ116347 | A/TAI/19/2008 | Sea-97 (G3) | 2008 | Thailand | cattle |
| HQ116348 | A/TAI/5/2009 | Sea-97 (G3) | 2009 | Thailand | cattle |
| HQ116349 | A/TAI/6/2009 | Sea-97 (G3) | 2009 | Thailand | cattle |
| HQ116350 | A/TAI/7/2009 | Sea-97 (G3) | 2009 | Thailand | cattle |
| HQ116351 | A/TAI/8/2009 | Sea-97 (G3) | 2009 | Thailand | cattle |
| HQ116352 | A/TAI/9/2009 | Sea-97 (G3) | 2009 | Thailand | cattle |
| HQ116353 | A/TAI/10/2009 | Sea-97 (G3) | 2009 | Thailand | cattle |
| HQ116354 | A/TAI/14/2009 | Sea-97 (G3) | 2009 | Thailand | cattle |
| HQ116355 | A/TAI/2/98 | Sea-97 (G1) | 1998 | Thailand | cattle |
| HQ116356 | A/TAI/1/99 | Sea-97 (G1) | 1999 | Thailand | cattle |
| HQ116357 | A/TAI/6/99 | Sea-97 (G1) | 1999 | Thailand | cattle |
| HQ116358 | A/VIT/4/2004 | Sea-97 (G2) | 2004 | Vietnam | cattle |
| HQ116359 | A/VIT/5/2004 | Sea-97 (G2) | 2004 | Vietnam | cattle |
| HQ116360 | A/VIT/6/2004 | Sea-97 (G2) | 2004 | Vietnam | cattle |
| HQ116361 | A/VIT/9/2004 | Sea-97 (G2) | 2004 | Vietnam | cattle |
| HQ116362 | A/VIT/10/2004 | Sea-97 (G2) | 2004 | Vietnam | cattle |
| HQ116363 | A/VIT/11/2004 | Sea-97 (G2) | 2004 | Vietnam | cattle |
| HQ116364 | A/VIT/12/2004 | Sea-97 (G2) | 2004 | Vietnam | cattle |
| HQ116365 | A/VIT/8/2005 | Sea-97 (G2) | 2005 | Vietnam | cattle |
| HQ116366 | A/VIT/10/2005 | Sea-97 (G2) | 2005 | Vietnam | cattle |
| HQ116367 | A/VIT/13/2005 | Sea-97 (G2) | 2005 | Vietnam | cattle |
| HQ116368 | A/VIT/14/2005 | Sea-97 (G2) | 2005 | Vietnam | cattle |
| HQ116369 | A/VIT/18/2005 | Sea-97 (G2) | 2005 | Vietnam | cattle |
| HQ116370 | A/VIT/2/2008 | Sea-97 (G3) | 2008 | Vietnam | cattle |
| HQ116371 | A/VIT/3/2008 | Sea-97 (G3) | 2008 | Vietnam | cattle |
| HQ116372 | A/VIT/4/2008 | Sea-97 (G3) | 2008 | Vietnam | buffalo |
| HQ116373 | A/VIT/5/2008 | Sea-97 (G3) | 2008 | Vietnam | cattle |
| HQ116374 | A/VIT/6/2008 | Sea-97 (G3) | 2008 | Vietnam | cattle |
| HQ116375 | A/VIT/7/2008 | Sea-97 (G3) | 2008 | Vietnam | buffalo |
| HQ116376 | A/VIT/8/2008 | Sea-97 (G3) | 2008 | Vietnam | cattle |
| HQ116377 | A/VIT/1/2009 | Sea-97 (G3) | 2009 | Vietnam | cattle |
| HQ116378 | A/VIT/2/2009 | Sea-97 (G3) | 2009 | Vietnam | buffalo |
| HQ116379 | A/VIT/3/2009 | Sea-97 (G3) | 2009 | Vietnam | cattle |
| HQ116380 | A/VIT/4/2009 | Sea-97 (G3) | 2009 | Vietnam | cattle |
| HQ116381 | A/VIT/5/2009 | Sea-97 (G3) | 2009 | Vietnam | buffalo |
| HQ116382 | A/VIT/6/2009 | Sea-97 (G3) | 2009 | Vietnam | pig |
| HQ116383 | A/VIT/7/2009 | Sea-97 (G3) | 2009 | Vietnam | buffalo |
| HQ116384 | A/VIT/8/2009 | Sea-97 (G3) | 2009 | Vietnam | buffalo |
| JF792355 | A/HuBWH/CHA/2009 | Sea-97 (G3) | 2009 | China | cattle |
| JN006722 | A/SIN/PAK/L4/2008 | Iran-05 | 2008 | Pakistan | cattle |
| JQ070331 | A/SKR/2/2010 | Sea-97 (G3) | 2010 | South Korea | cattle |
| JQ070332 | A/VIT/1/2010 | Sea-97 (G3) | 2010 | Vietnam | cattle |
| JX435106 | A/BAL/PAK/ISO2/2011 | Iran-05 | 2011 | Pakistan | cattle |
| JX435107 | A/BAL/PAK/ISO3/2011 | Iran-05 | 2011 | Pakistan | cattle |
| JX435108 | A/BAL/PAK/ISO5/2011 | Iran-05 | 2011 | Pakistan | cattle |
| JX462599 | A/BAL/PAK/ISO1/2011 | Iran-05 | 2011 | Pakistan | cattle |
| KF450794 | A/GDMM/CHA/2013 | Sea-97 (G4) | 2013 | China | pig |
| KJ608371 | A/VN/T11D/2013 | Sea-97 (G4) | 2013 | Vietnam | cattle |
| KT832824 | A/BAL/PAK/05/2011 | Iran-05 | 2011 | Pakistan | cattle |
| KT832825 | A/BAL/PAK/ISO4/2011 | Iran-05 | 2011 | Pakistan | cattle |
| KT832827 | A/BAL/PAK/12/2011 | Iran-05 | 2011 | Pakistan | cattle |
| KT832829 | A/BAL/PAK/19/2011 | Iran-05 | 2011 | Pakistan | cattle |
| KT832830 | A/BAL/PAK/21/2011 | Iran-05 | 2011 | Pakistan | cattle |
| KT832832 | A/BAL/PAK/22/2011 | Iran-05 | 2011 | Pakistan | cattle |
| KT832834 | A/BAL/PAK/23/2011 | Iran-05 | 2011 | Pakistan | cattle |
| KT832835 | A/BAL/PAK/ISO7/2011 | Iran-05 | 2011 | Pakistan | cattle |
| KT832836 | A/BAL/PAK/ISO8/2011 | Iran-05 | 2011 | Pakistan | cattle |
| KT832837 | A/BAL/PAK/30/2011 | Iran-05 | 2011 | Pakistan | cattle |
| KT832838 | A/BAL/PAK/30a/2011 | Iran-05 | 2011 | Pakistan | cattle |
| KT832839 | A/BAL/PAK/34/2011 | Iran-05 | 2011 | Pakistan | cattle |
| KT832840 | A/BAL/PAK/34a/2011 | Iran-05 | 2011 | Pakistan | cattle |
| KT968663 | A/HY/CHA/2013 | Sea-97 (G4) | 2013 | China | domestic yak |
| KY091290 | A/IRN/9/2010 | Iran-05 | 2010 | Iran | cattle |
| KY091291 | A/IRN/78/2009 | Iran-05 | 2009 | Iran | - |
| KY091292 | A/IRN/1/2011 | Iran-05 | 2011 | Iran | cattle |
| KY091293 | A/AFG/10/2010 | Iran-05 | 2010 | Afghanistan | - |
| KY091294 | A/TUR/3/2010 | Iran-05 | 2010 | Turkey | cattle |
| KY091295 | A/IRN/9/2011 | Iran-05 | 2011 | Iran | cattle |
| KY091296 | A/IRN/125/2010 | Iran-05 | 2010 | Iran | cattle |
| KY091297 | A/IRN/15/2012 | Iran-05 | 2012 | Iran | cattle |
| KY322675 | A/LAO/3/2014 | Sea-97 (G5) | 2014 | Laos | cattle |
| KY322676 | A/MAY/12/2013 | Sea-97 (G4) | 2013 | Malaysia | cattle |
| KY322677 | A/MAY/20/2013 | Sea-97 (G5) | 2013 | Malaysia | cattle |
| KY322678 | A/MAY/23/2013 | Sea-97 (G4) | 2013 | Malaysia | cattle |
| KY322679 | A/TAI/4/2014 | Sea-97 (G5) | 2014 | Thailand | cattle |
| KY322680 | A/VIT/42/2013 | Sea-97 (G5) | 2013 | Vietnam | cattle |
| KY766148 | A/YC/SKR/2017 | Sea-97 (G4) | 2017 | South Korea | cattle |
| KY780958 | A/PD78/IND/2015 | G-VII | 2015 | India | cattle |
| KY982279 | A/ARM/1/2015 | G-VII | 2015 | Armenia | cattle |
| KY982280 | A/ARM/2/2015 | G-VII | 2015 | Armenia | cattle |
| KY982281 | A/ARM/3/2015 | G-VII | 2015 | Armenia | cattle |
| KY982282 | A/IRN/8/2015 | G-VII | 2015 | Iran | cattle |
| KY982283 | A/IRN/12/2015 | G-VII | 2015 | Iran | cattle |
| KY982284 | A/IRN/13/2015 | G-VII | 2015 | Iran | cattle |
| KY982285 | A/IRN/14/2015 | G-VII | 2015 | Iran | cattle |
| KY982286 | A/IRN/17/2015 | G-VII | 2015 | Iran | cattle |
| KY982287 | A/IRN/18/2015 | G-VII | 2015 | Iran | cattle |
| KY982288 | A/IRN/21/2015 | G-VII | 2015 | Iran | cattle |
| KY982289 | A/IRN/22/2015 | G-VII | 2015 | Iran | cattle |
| KY982290 | A/IRN/25/2015 | G-VII | 2015 | Iran | cattle |
| KY982291 | A/IRN/27/2015 | G-VII | 2015 | Iran | cattle |
| KY982292 | A/IRN/1/2016 | G-VII | 2016 | Iran | cattle |
| KY982293 | A/IRN/8/2016 | G-VII | 2016 | Iran | cattle |
| KY982294 | A/IRN/11/2016 | G-VII | 2016 | Iran | cattle |
| KY982295 | A/IRN/12/2016 | G-VII | 2016 | Iran | cattle |
| KY982296 | A/IRN/20/2016 | G-VII | 2016 | Iran | cattle |
| KY982297 | A/IRN/23/2016 | G-VII | 2016 | Iran | cattle |
| KY982298 | A/SAU/2/2015 | G-VII | 2015 | Saudi Arabia | cattle |
| KY982299 | A/SAU/3/2015 | G-VII | 2015 | Saudi Arabia | cattle |
| KY982300 | A/SAU/4/2015 | G-VII | 2015 | Saudi Arabia | cattle |
| KY982301 | A/SAU/5/2015 | G-VII | 2015 | Saudi Arabia | cattle |
| KY982302 | A/SAU/6/2015 | G-VII | 2015 | Saudi Arabia | cattle |
| KY982303 | A/SAU/7/2015 | G-VII | 2015 | Saudi Arabia | cattle |
| KY982304 | A/SAU/8/2015 | G-VII | 2015 | Saudi Arabia | cattle |
| KY982305 | A/SAU/9/2015 | G-VII | 2015 | Saudi Arabia | cattle |
| KY982306 | A/SAU/14/2015 | G-VII | 2015 | Saudi Arabia | sheep |
| KY982307 | A/SAU/15/2015 | G-VII | 2015 | Saudi Arabia | sheep |
| KY982308 | A/SAU/16/2015 | G-VII | 2015 | Saudi Arabia | sheep |
| KY982309 | A/SAU/17/2015 | G-VII | 2015 | Saudi Arabia | sheep |
| KY982310 | A/SAU/21/2015 | G-VII | 2015 | Saudi Arabia | cattle |
| KY982311 | A/SAU/15/2016 | G-VII | 2016 | Saudi Arabia | cattle |
| KY982312 | A/SAU/19/2016 | G-VII | 2016 | Saudi Arabia | cattle |
| KY982313 | A/SAU/20/2016 | G-VII | 2016 | Saudi Arabia | cattle |
| KY982314 | A/SAU/21/2016 | G-VII | 2016 | Saudi Arabia | cattle |
| KY982315 | A/SAU/22/2016 | G-VII | 2016 | Saudi Arabia | cattle |
| KY982316 | A/SAU/24/2016 | G-VII | 2016 | Saudi Arabia | cattle |
| KY982317 | A/SAU/37/2016 | G-VII | 2016 | Saudi Arabia | cattle |
| KY982318 | A/SAU/40/2016 | G-VII | 2016 | Saudi Arabia | cattle |
| KY982319 | A/SAU/41/2016 | G-VII | 2016 | Saudi Arabia | cattle |
| KY982320 | A/SAU/42/2016 | G-VII | 2016 | Saudi Arabia | cattle |
| KY982321 | A/TUR/175/2015.712 | G-VII | 2015 | Turkey | cattle |
| KY982322 | A/TUR/198/2015.808 | G-VII | 2015 | Turkey | cattle |
| KY982323 | A/TUR/203/2015.827 | G-VII | 2015 | Turkey | cattle |
| KY982324 | A/TUR/219/2015.865 | G-VII | 2015 | Turkey | cattle |
| KY982325 | A/TUR/305/2015.923 | G-VII | 2015 | Turkey | cattle |
| KY982326 | A/TUR/331/2015.923 | G-VII | 2015 | Turkey | cattle |
| KY982327 | A/TUR/48/2016.019 | G-VII | 2016 | Turkey | sheep |
| KY982328 | A/TUR/1008/2016.500 | G-VII | 2016 | Turkey | cattle |
| KY982329 | A/TUR/1193/2016.731 | G-VII | 2016 | Turkey | cattle |
| KY982330 | A/TUR/1210/2016.750 | G-VII | 2016 | Turkey | cattle |
| KY982331 | A/TUR/1218/2016.769 | G-VII | 2016 | Turkey | cattle |
| KY982332 | A/TUR/1225/2016.769 | G-VII | 2016 | Turkey | cattle |
| KY982333 | A/TUR/1227/2016.750 | G-VII | 2016 | Turkey | cattle |
| LC483874 | A/TAI/SEA-97/2007 | Sea-97 (G2) | 2007 | Thailand | cattle |
| MF947133 | A/VIT/12/2010 | Sea-97 (G3) | 2010 | Vietnam | cattle |
| MG552837 | A/Egy/Beheira/2015 | Iran-05 | 2015 | Egypt | cattle |
| MG552838 | A/Egy/Damietta/2015 | Iran-05 | 2015 | Egypt | buffalo |
| MG552839 | A/Egy/Monufia/2015 | Iran-05 | 2015 | Egypt | buffalo |
| MG552840 | A/Egy/Qalyubia/2015 | Iran-05 | 2015 | Egypt | buffalo |
| MG552841 | A/Egy/Beni_Suef/2016 | Iran-05 | 2016 | Egypt | cattle |
| MG552842 | A/Egy/Damietta/2016 | Iran-05 | 2016 | Egypt | cattle |
| MG840802 | A/GZCS/CHA/2018 | Sea-97 (G4) | 2018 | China | cattle |
