# Supplementary material for: Phylogenetic and evolutionary analysis of foot-and-mouth disease virus A/ASIA/Sea-97 lineage
Source: Virus Genes. 2021 Jul 14;57(5):443–7. doi: 10.1007/s11262-021-01848-7 (PMC8445868; doi:10.1007/s11262-021-01848-7)
Supplement: Supplementary file 3 — (DOCX 17 kb) [file 11262_2021_1848_MOESM3_ESM.docx]

**Phylogenetic and Evolutionary Analysis of Foot-and-Mouth Disease Virus A/ASIA/Sea-97 Lineage**

Soyeon Bae^1^, Vladimir Li^2^, Juyong Hong^1^, Jin Nam Kim^3^ and Heebal Kim^1,2,3,*^

^1^Department of Agricultural Biotechnology and Research Institute of Agriculture and Life Sciences, Seoul National University, Seoul 08826, Republic of Korea.

^2^Interdisciplinary Program in Bioinformatics, Seoul National University, Seoul 08826, Republic of Korea.

^3^eGnome, Inc, Seoul, Republic of Korea.

^*^Corresponding Author: Heebal Kim [heebal@snu.ac.kr](mailto:heebal@snu.ac.kr)

**Table S2** Selection analysis of FMDV A/ASIA/Sea-97

|  | **MEME**  **(p-value < 0.1)** | **FEL**  **(p-value < 0.1)** | **FUBAR**  **(posterior probability > 0.9)** | **SLAC**  **(p-value < 0.1)** |
| --- | --- | --- | --- | --- |
| **Sites under positive selection** | 59, 85, 96, 141, 142, 149, 207 | 96, 141, 142, 149, 173 | 45, 141, 142, 149, 173 | 45, 99, 141, 142, 149 |
